# Thermal heterogeneity, migration, and consequences for spawning potential of female bull trout in a river–reservoir system

**DOI:** 10.1002/ece3.6184

**Published:** 2020-04-03

**Authors:** Joseph R. Benjamin, Dmitri T. Vidergar, Jason B. Dunham

**Affiliations:** ^1^ U.S. Geological Survey Forest and Rangeland Ecosystem Science Center Boise Idaho; ^2^ Bureau of Reclamation Snake River Area Office Boise Idaho; ^3^ U.S. Geological Survey Forest and Rangeland Ecosystem Science Center Corvallis Oregon

**Keywords:** bioenergetics, Boise River, energy content, migration, *Salvelinus confluentus*, spawning, temperature

## Abstract

The likelihood that fish will initiate spawning, spawn successfully, or skip spawning in a given year is conditioned in part on availability of energy reserves. We evaluated the consequences of spatial heterogeneity in thermal conditions on the energy accumulation and spawning potential of migratory bull trout (*Salvelinus confluentus*) in a regulated river–reservoir system. Based on existing data, we identified a portfolio of thermal exposures and migratory patterns and then estimated their influence on energy reserves of female bull trout with a bioenergetics model. Spawning by females was assumed to be possible if postspawning energy reserves equaled or exceeded 4 kJ/g. Given this assumption, results suggested up to 70% of the simulated fish could spawn each year. Fish that moved seasonally between a cold river segment and a warmer reservoir downstream had a greater growth rate and higher propensity to spawn in a given year (range: 40%–70%) compared with fish that resided solely in the cold river segment (25%–40%). On average, fish that spawned lost 30% of their energy content relative to their prespawn energy. In contrast, fish that skipped spawning accumulated, on average, 16% energy gains that could be used toward future gamete production. Skipped spawning occurred when water temperatures were relatively low or high, and if upstream migration occurred relatively late (mid‐July or later) or early (early‐May or earlier). Overall, our modeling effort suggests the configuration of thermal exposures, and the ability of bull trout to exploit this spatially and temporally variable thermal conditions can strongly influence energy reserves and likelihood of successful spawning.

## Graphical abstract

We evaluated the consequences of spatial heterogeneity in thermal conditions on the energy accumulation and spawning potential of migratory bull trout (*Salvelinus confluentus*) in a regulated river–reservoir system. Overall, our modeling effort suggests the configuration of thermal exposures and the ability of bull trout to exploit this spatially and temporally variable thermal conditions can strongly influence energy reserves and likelihood of successful spawning.

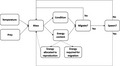

## 1 INTRODUCTION

Thermal heterogeneity in space and time provides a template of opportunity and constraint for fishes migrating through river networks (Fullerton et al., 2018; Snyder et al., 2019). In the case of coldwater species such as salmonids, constraints are imposed by water temperatures that are unsuitably warm for growth, reproduction, or survival (McCullough et al., 2009). Such constraints are increasing in frequency and duration as water temperatures warm in response to changing climates (Jonsson & Jonsson, 2009; Kovach et al., 2016) and altered by river regulation (Brekke et al., 2009; Johnson et al., 2004). In many circumstances, rather than acting as a constraint, thermally heterogeneous environments can provide opportunities for fish to behaviorally thermoregulate and select locations that maximize growth, reproduction, and survival (Fullerton et al., 2018; Hughes & Grand, 2000; Mehner, 2012). Thus, the question of whether changing thermal heterogeneity is an asset or a liability depends on (a) the ability of fish to detect and access thermal resources (Magnuson, Crowder, & Medvick, 1979; Nathan et al., 2008), and the (b) consequences of thermal habitat use for growth, reproduction, and survival (Fullerton et al., 2018; McCullough et al., 2009; Snyder et al., 2019).

Here, we evaluate the consequences of alternative patterns of spatial and seasonal thermal habitat use by bull trout (*Salvelinus confluentus*) in a highly regulated river–reservoir system. Bull trout is native to the eastern Pacific Rim of North America (Dunham et al., 2008), and among the most cold‐adapted of all aquatic vertebrates in the region (Benjamin, Heltzel, Dunham, Heck, & Banish, 2016; Dunham, Rieman, & Chandler, 2003; Isaak, Wenger, & Young, 2017). Patterns of thermal habitat use have been studied directly or indirectly via telemetry within rivers (e.g., Howell, Dunham, & Sankovich, 2010; Paragamian & Walters, 2011; Swanberg, 1997) and lakes and reservoirs (e.g., Eckmann, Dunham, Connor, & Welch, 2018; Gutowsky et al., 2017), but there are no studies of the consequences of thermal habitat use by individuals moving through these linked river–reservoir systems. Our overall goal in this study was to address this gap by conducting an integrated analysis of thermal habitat use by bull trout moving through linked river–reservoir systems.

**Figure 1:**
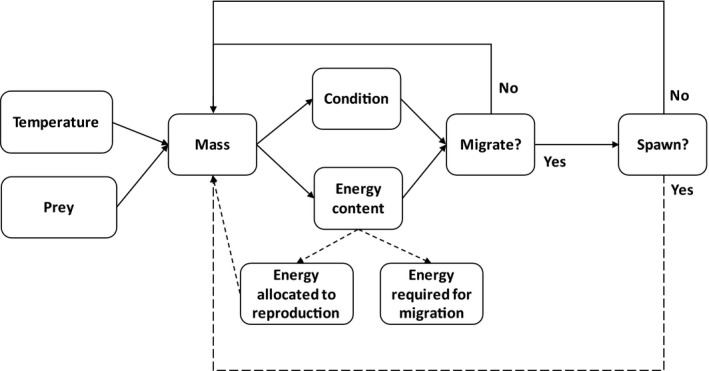
Conceptual diagram of the linkages from bioenergetics inputs (temperature, prey quality, and quantity) to estimate mass (g), energy content (kJ/g), and condition factor of female bull trout and ultimately the potential of migrating to spawning grounds and spawning. Dashed lines indicate a loss in energy or mass based on the trajectory exhibited

In this study, we employed a bioenergetics approach (Deslauriers, Chipps, Breck, Rice, & Madenjian, 2017; Mesa, Weiland, Christiansen, Sauter, & Beauchamp, 2013) to estimate energy available for reproduction and understand the potential consequences of thermal habitat use in a regulated river–reservoir system. As fish move through stream networks, energy is expended through physiological costs linked to temperature, maturation, movement, and body size (Figure 1). These costs are balanced with gains from consumption. Fish with sufficient energy are capable of migrating to spawning grounds and reproducing. However, if fish lack sufficient energy reserves, they may fail to migrate altogether or migrate and arrive at spawning destinations without enough energy to reproduce (Jørgensen, Ernande, Fiksen, & Dieckmann, 2006; Rideout, Rose, & Burton, 2005; Figure 1). Either outcome leads to skipped spawning in a given year. Energetic costs were modeled based on scenarios involving contrasting thermal exposures constructed from observed migratory behavior of bull trout in the river–reservoir system we studied. Modeling was focused on the specific requirements of females (due to their greater investment in gonads; Jørgensen et al., 2006; Rideout et al., 2005), and for all scenarios, we tracked growth, energy content, condition, age of first spawning, and frequency of repeated (or skipped) spawning. Results of this work build on previous work linking energy content to spawning potential of other fishes (e.g., Glebe & Leggett, 1981; Jørgensen et al., 2006; Plumb, 2018; Plumb, Blanchfield, & Abrahams, 2014), as well as insights on the prevalence of skipped spawning in iteroparous fishes (Bull & Shine, 1979; Rideout et al., 2005; Secor, 2008) to provide an integrated modeling framework for evaluating the reproductive consequences of alternative patterns of habitat use by bull trout in a river–reservoir system.

## 2 METHODS

### 2.1 Study area

**Figure 2:**
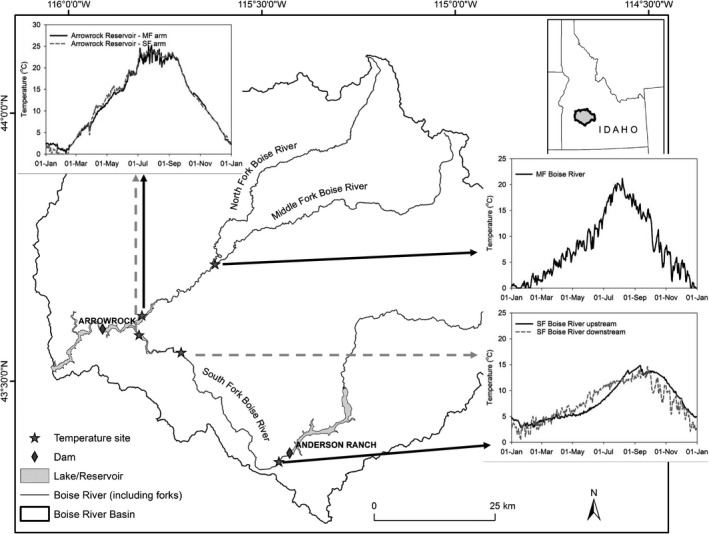
Map of the upper Boise River basin. The temperature sites and daily average temperature (°C) used in model simulations for different sections fish occupy in the upper Boise River basin

We focused our model simulations of bull trout spawning success in the upper Boise River, Idaho, which is near the southernmost limit of bull trout's native range (Figure 2). The upper Boise River offers a diverse hydrologic template and includes three sub‐basins (North, Middle, and South Forks) and two dams (Arrowrock Dam and Anderson Ranch Dam). Anderson Ranch Reservoir and upstream are also part of the upper Boise River basin, but not considered in the current study because the population above Anderson Ranch Dam is isolated from the population below. The basin drains mountainous terrain that is largely comprised of Idaho Batholith; as such, the geology can be highly erosive with extensive sediment deposits near the upper extent of Arrowrock Reservoir. Elevations in the basin range from approximately 945 m to 3,231 m. Winter climate is typically cold (minimum air temperature below 0°C), and summer, warm (>30°C). A typical snowmelt discharge regime is exhibited with peak flows occurring in the spring and baseflows in late summer.

Downstream of Anderson Ranch Reservoir is Arrowrock Reservoir, which is managed for irrigation and flood control by the Bureau of Reclamation. Arrowrock Reservoir is the most downstream habitat available to bull trout in the upper Boise River basin (Monnot, Dunham, Hoem, & Koetsier, 2008). This highly regulated reservoir typically releases about 86% of its volume annually (maximum pool volume = 0.336 km^3^), with a goal to maintain more than 0.047 km^3^ throughout the year for fish habitat. However, cold thermal refugia for bull trout during August and September, when reservoir volume is at the lowest level of the year, may be limited because water temperatures often exceed 15°C and dissolved oxygen can be below 6.5 mg/l (Maret & Schultz, 2013).

Temperature and discharge in the SF Boise River are regulated by operation of Anderson Ranch Dam, which is managed to release cold water from the hypolimnion of Anderson Ranch Reservoir (Benjankar et al., 2018; Figure 2). Water releases typically peak in the spring and summer months when demands for irrigation are greater. Little is known about how thermal heterogeneity influences bull trout within the reservoir and SF Boise River. However, seasonal movement patterns of bull trout have been described (Flatter, 2000; Monnot et al., 2008; Salow & Hostettler, 2004) and critical habitat for foraging, migration, and overwintering identified (U.S. Fish & Wildlife Service, 2005, 2015), which requires evaluation of effects of management actions on bull trout and their habitat.

Bull trout in the upper Boise River basin display life history strategies similar to many other populations with access to larger riverine, lacustrine, or marine habitats (Al‐Chokhachy & Budy, 2008; Brenkman, Corbett, & Volk, 2007; Johnston & Post, 2009). Spawning during September through October and juvenile rearing occur in the headwaters of the NF and MF Boise River (Dunham & Rieman, 1999; Monnot et al., 2008). A portion of the population remains near these headwaters for the duration of their life (referred to as a resident life history), whereas others migrate to Arrowrock Reservoir or the SF Boise River (migratory life history; Maret & Schultz, 2013; Monnot et al., 2008).

### 2.2 Bull trout movement patterns

To evaluate the migratory behaviors displayed by bull trout in the Boise River basin, we used previous telemetry observations (Flatter, 2000; MacCoy, Shephard, Benjamin, Vidergar, & Prisciandaro, 2017; Maret & Schultz, 2013; Monnot et al., 2008; Salow & Hostettler, 2004). Most migratory bull trout overwinter (October to March) in Arrowrock Reservoir, return to the MF or NF Boise River between March and July, remain in the headwaters until fall spawning, and then return to Arrowrock Reservoir. However, approximately 25% of adult bull trout reside in the SF Boise River during a portion of their life (Salow & Hostettler, 2004), which was the focus of this study. In general, bull trout that occupied the SF Boise River exhibited four different movement patterns. First, migratory bull trout would use the SF Boise River to overwinter, then move to the MF/NF Boise River in the spring through spawning. Second, bull trout occupy Arrowrock Reservoir in fall (mid‐September to mid‐November) and winter, migrate upstream into the SF Boise River in spring (March‐July), remain in the SF Boise River during summer, and then migrate to the reservoir again to overwinter. Third, bull trout reside in the SF throughout the year before migrating to the headwaters of the NF and MF Boise River to spawn. Fourth, bull trout overwinter in Arrowrock Reservoir and periodically move between Arrowrock Reservoir and the SF Boise River during summer. Any of these patterns can happen for one or more years without completing a spawning migration to the headwaters of the NF or MF Boise River. Spawning is not known to occur in the mainstem of the SF Boise River.

### 2.3 Bioenergetics model

We used a bioenergetics model (Deslauriers et al., 2017; Hanson, Johnson, Schindler, & Kitchell, 1997) and physiological parameters for bull trout (Mesa et al., 2013) to explore the mass (g) and energy content (kJ/g) of female bull trout displaying different movement patterns in the SF Boise River. Mass of an individual under the bioenergetics model was determined by daily growth rates influenced by the physiology of the fish, water temperature, and food quality and availability. Growth is estimated as the difference of energy consumed from energy needed for metabolic cost (i.e., respiration, digestion) and waste (i.e., excretion and egestion), all of which are temperature‐dependent via exponential functions (Hanson et al., 1997; Mesa et al., 2013). We focused on females because, relative to males, more reproductive investment is needed for egg development (Jonsson, Jonsson, & Hansen, 1991, 1997; Jørgensen et al., 2006) and females are more likely to exhibit migratory behaviors (Kendall et al., 2015).

#### 2.3.1 Water temperature

The average daily water temperature needed for the bioenergetics model was calculated from empirical data or estimated from model simulations. We used average temperatures because the daily variance is lower in colder streams (Dunham, Chandler, Rieman, & Martin, 2005), like the SF Boise River, and bull trout do not appear to use available thermal refuges, at least where this has been studied in detail for migratory individuals (Howell et al., 2010). Moreover, these average temperatures used for simulations were similar to temperature use by three bull trout observed from temperature sensory telemetry and archival tags (see geodatabase described in MacCoy et al., 2017). In the SF Boise River, the average daily water temperature was collected at stream gauges maintained by the U.S. Geological Survey (USGS; downloaded at http://waterdata.usgs.gov) just above Arrowrock Reservoir (site No. 13192200 from May 2011 to December 2017) and below Anderson Ranch Dam (from February 2013 to November 2014 by U.S. Bureau of Reclamation (USBR) and site No. 13190500, from January to December 2017) (Figure 2). We considered these two locations to represent the lower and upper segments, respectively, of the SF Boise River. For Arrowrock Reservoir, we used daily temperatures simulated from a two‐dimensional, hydrodynamic, and water quality model (Cole & Wells, 2017) modified for Arrowrock Reservoir (Bureau of Reclamation, 2018). The water quality model is well suited for lakes, reservoirs, and other water bodies that thermally stratify and can simulate water temperature, dissolved oxygen, nutrients, and other water quality metric at multiple depths. We identified two locations in Arrowrock Reservoir for estimated temperature values, one in each of the south and north arm (Figure 2). We averaged daily temperatures across water depths between 0 and 10 m because the average depth experienced by a bull trout with acoustic telemetry tags in Arrowrock Reservoir was 6.7 m and over 80% of fish detected were at a depth of <10 m (Maret & Schultz, 2013). Thermal stratification occurs from approximately June through August, but, for most of the water column, temperatures and dissolved oxygen remain within suitable ranges for bull trout (Maret & Schultz, 2013). Moreover, bull trout were not observed in the reservoir during these months (Maret & Schultz, 2013). Thus, we opted not to consider the consequences of stratification for bull trout. In addition to locations in the SF Boise River and Arrowrock Reservoir, we used temperatures collected daily during 2012 in the MF Boise River to account for the time fish spent when migrating to spawning grounds (MacCoy et al., 2017). When multiple year data were available, we averaged the daily water temperature across years to develop an average annual temperature cycle for each site, which was used for each year in the bioenergetics model.

#### 2.3.2 Food quality and quantity

To estimate the quality of food consumed by bull trout, we used data from an analysis of the prey items of 50 bull trout (range: 62–4,550 g). Stomach contents were collected using gastric lavage during May 2012 from fish captured in Arrowrock Reservoir. Twenty‐five (50%) of the fish sampled had empty stomachs. Of the remaining 25 fish with contents in their stomachs, fish made up the largest percentage (98%) by weight of the dietary contents and was dominated by yellow perch (*Perca flavescens*) and salmonids (Salmonidae). Bull trout appeared to track the fish species with greater relative abundance (MacCoy et al., 2017), which is similar to bull trout in other systems (Beauchamp & Van Tassell, 2001; Fraley & Shepard, 1989; Lowery & Beauchamp, 2015). Based on data from the diet contents and quarterly fish surveys from 2011 to 2014 (MacCoy et al., 2017), we estimated a range of energy densities from approximately 4,000 J/g to 6,000 J/g, which include fish and invertebrates (Beauchamp & Van Tassell, 2001; Hanson et al., 1997). To account for uncertainty in the quality of the prey being consumed, in model simulations we randomly assigned daily prey energy density using a normal distribution (Mean = 5,000 J/g; *SD* = 1,270 J/g).

The proportion of maximum consumption (*P*_Cmax_), a surrogate for food availability, was estimated by fitting a separate bioenergetics model to observed growth of marked–recaptured migratory bull trout (*n* = 11; marked fish (mean ± *SE*): 1,169 ± 244 g; recaptured fish: 1,433 ± 271 g; MacCoy et al., 2017). For the *P*_Cmax_ estimate, we used the average temperatures and energy density of prey fish described above. Some bull trout occupied more than one water segment (e.g., upper and lower SF Boise River; different arms of Arrowrock Reservoir). We attempted to estimate *P*_Cmax_ by sections of the watershed based upon migration timing of radio‐tagged fish (MacCoy et al., 2017). We estimated mean *P*_Cmax_ for Arrowrock Reservoir (*P*_Cmax_ = 0.26; *SD* = 0.14; *n* = 6), for SF Boise River (mean *P*_Cmax_ = 0.22; *SD* = 0.05; *n* = 4), and for MF Boise River (mean *P*_Cmax_ = 0.19; assumed *SD* = 0.05; *n* = 1). The latter was used for fish during spawning migrations. We structured the model to randomly assign a different daily *P*_Cmax_ for each fish in each watershed section, drawn from a normal distribution using the means and *SD* above.

### 2.4 Energy content and allocation

Energy content and allocation for individual fish was estimated from empirical equations. First, energy density (*ED*; J/g), as a function of weight (*W*; g) at time *t*, was calculated at a daily time step from a regression equation in Mesa et al. (2013; *ED* = 6,410 + 0.367*W_t_*). Second, energy content (*EC*; J) of a simulated fish at time *t* was estimated as the product of the fish's energy density and mass.

We assumed energy content is allocated to either somatic growth or reproduction. To separate mass and energy associated with fish growth, we first estimated the mass allocated to somatic growth by subtracting the mass of the gonads from the total mass provided by the biogenetics model. This step was necessary because the Wisconsin bioenergetics model does not separate somatic and gonadal mass. We assumed 17.1% of accrued total energy content would be allocated to gonads, which is consistent with gonadosomatic index values for female Dolly Varden (Armstrong & Bond, 2013), Arctic charr (Finstad, Berg, Langeland, & Lohrmann, 2002), and other female salmonids (Fleming, 1998). Once separated, we calculated energy content available for growth and reproduction.

During migration to spawning grounds, approximately 4,000 J/km of energy was assumed to be used based on values calculated for Atlantic and sockeye salmon (Crossin et al., 2004; Jonsson, Jonsson, & Hansen, 1997; Rand et al., 2006). The average one‐way distance a bull trout migrates from the SF Boise River to the spawning grounds in the headwaters of the NF and MF Boise River was assumed to be 100 km. Thus, a total of 400 kJ was subtracted from the total energy content. We assumed the energetic cost of downstream migration would be minimal relative to the cost of upstream migration and did not include an energetic cost for this event. Energy allocated to reproduction and required for spawning was subtracted from the total energy to estimate the amount of energy remaining if a fish spawns (see below).

### 2.5 Migration and spawning rules

We assumed that successful spawning by female bull trout requires sufficient available energy to allocate to reproductive development, migration, and the act of spawning, with enough energy reserves remaining to return to habitats used for feeding, refuge, overwintering, or other nonreproductive purposes. Empirical estimates are not available for the minimal energy content threshold for successful spawning of bull trout, but Dutil (1986) reported that postspawning Arctic charr, a congener of bull trout, had approximately 4–5 kJ/g of energy reserves upon returning to downstream rearing habitat. We therefore assumed an adult migratory female bull trout would need at least 4 kJ/g of energy content reserved postspawning. If the 4 kJ/g threshold value could not be maintained, then successful migration and spawning did not occur, and the individual skipped spawning for that year.

At the time of migration, if an individual did not have 4 kJ/g of energy reserves after accounting for migration and spawning costs, then the individual would not migrate. In addition, to a minimal energy content, a successfully migrating fish was assumed to have a healthy condition factor (CF), which we defined as 0.9 or greater. Because length is required to calculate CF, we estimated length (*L*) of the individual from the mass following equations in Railsback, Harvey, Jackson, and Lamberson (2005). For a length–weight relationship, we used empirically derived data for migratory bull trout in the Boise River (*W_g_* = 0.000003 * *L* ^3.1993^), where *W_g_* is weight in g, and *L* is fork length in mm. We assumed length of a fish can increase over time, but will never be reduced, even if the mass does. For example, if a fish is 500 mm and 1,300 g on day *t* and 1,200 g thirty days later, the length of the fish will remain at 500 mm. On average, timing of upstream migration to spawning grounds was on 01 June. Downstream migration was on 01 October (sensu MacCoy et al., 2017; Monnot et al., 2008).

Following a successful migration to spawning grounds, an adult female bull trout may successfully spawn, which was set at 01 September, the median spawning date. Similar to migration, at the time of spawning an individual must have 4 kJ/g of energy remaining after accounting for migration and spawning costs. If not, then the fish would not spawn. If the fish does not spawn owing to the lack of sufficient energy, we assumed eggs would be resorbed and energy preserved (Contreras‐Sánchez, Schreck, Fitzpatrick, & Pereira, 1998).

Postspawning, we assumed fish would lose mass owing to release of eggs, as well as other factors that influence the bioenergetics model (e.g., food resources, temperature). We estimated the amount of weight loss would be 89% of the mass allocated to reproductive development based on mass of Arctic charr pre‐ and postspawning (Dutil, 1986).

### 2.6 Model simulations

We started the model simulations with 1,000 subadult migratory female bull trout with a starting size of 250 g (approximately age‐2) that first enter Arrowrock Reservoir on 01 October, corresponding to field observations for size and migration timing (MacCoy et al., 2017; Monnot et al., 2008). Bull trout that exhibit a migratory life history may grow for an additional 7 years after first migrating to the reservoir, for a total of a 9‐year life span, which is the maximum age estimated for bull trout in the Boise River basin. We used model simulations as hypothetical examples to explore the growth potential of bull trout in different sections occupied and the potential to migrate and spawn. We assumed no mortality and that adult fish spawn each year if minimum criteria were met for migration and spawning.

**Figure 3:**
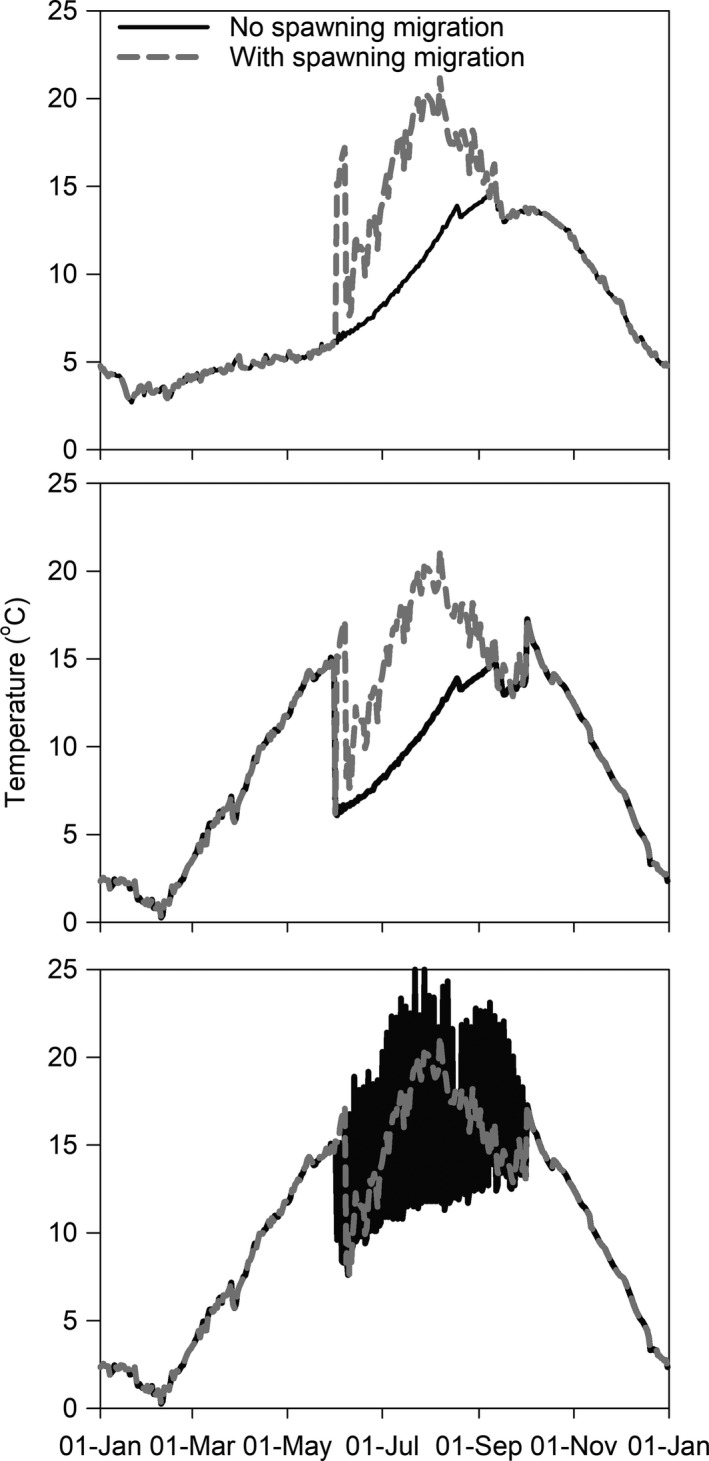
Example of temperature experienced by fish in movement scenarios 1 (top), 3 (middle), and 5 (bottom) during a year with and without a spawning migration. See Table 1 for movement scenario descriptions

For the purposes of model simulation, we simplified the movement patterns exhibited by bull trout (MacCoy et al., 2017; Monnot et al., 2008) into five movement scenarios (Table 1) and temperatures experienced (Figure 3). Bull trout will occupy the following: (a) the SF Boise River near Anderson Ranch Dam year‐round until a spawning migration occurs; (b) the lower SF Boise River above Arrowrock Reservoir year‐round until a spawning migration occurs; (c and d) Arrowrock Reservoir from mid‐fall through winter and SF Boise River (either near Anderson Ranch Reservoir or the lower section, respectively) during spring and summer; and (e) Arrowrock Reservoir from mid‐fall through winter, and during spring and summer, bull trout will move between the reservoir and SF Boise River over a 3‐day continuous cycle, which we assume will be 1 day in the reservoir and 2 days in the SF Boise River. These patterns repeat each year unless a fish migrates and spawns.

**Table 1:** General movement scenarios used in model simulations based on patterns exhibited by bull trout from telemetry studies

Movement scenario	Months		
	January–May	June–September	October–December
1	SF up	SF up	SF up
2	SF low	SF low	SF low
3	ARK	SF up	ARK
4	ARK	SF low	ARK
5	ARK	SF low/ARK^a^	ARK

Note

We randomly allocated the day (between 01 April and 01 August) of upstream migration into the SF Boise River using a uniform distribution; similar for the day (between 15 September and 20 November) of downstream migration to the reservoir. ARK = Arrowrock Reservoir, SF up = upstream location of SF Boise River near Anderson Ranch Dam, SF low = lower SF Boise River near Arrowrock Reservoir.

^a^ We assumed fish spend 1 day in Arrowrock Reservoir and 2 days in the lower SF Boise River.

We applied a random allocation from a uniform distribution for movements between Arrowrock Reservoir and the SF Boise River or during the spawning migration to the MF Boise River based on dates movement was observed in telemetry studies (MacCoy et al., 2017; Monnot et al., 2008). Movement upstream into the river was between 01 April and 01 August; these dates were also used for the migration to spawning grounds. Movement from the SF Boise River or spawning grounds to the reservoir was between 15 September and 19 November.

If a fish does migrate, we assumed it would take 7 days to swim through Arrowrock Reservoir and reach the MF Boise River site (Figure 2; MacCoy et al., 2017). During this time, fish are exposed to temperatures in the MF arm of Arrowrock Reservoir. After this time, fish will be exposed to temperatures in the MF Boise River. If enough energy remains for a fish to spawn, we assumed consumption (*P*_Cmax_) would be reduced by 50% 2 weeks (14 days) prior to 01 September, day of spawning, because of redd building and the act of spawning.

To account for potential environmental variation that could influence the growth of fish within the model, we randomly assigned a multiplier between −5% and 5% to daily temperature and prey quality (energy density of prey) and quantity (proportion of maximum consumption). Lastly, because of the uncertainty of what the threshold energy content was for bull trout to successfully migrate and spawn, we explored a range of thresholds.

## 3 RESULTS

**Figure 4:**
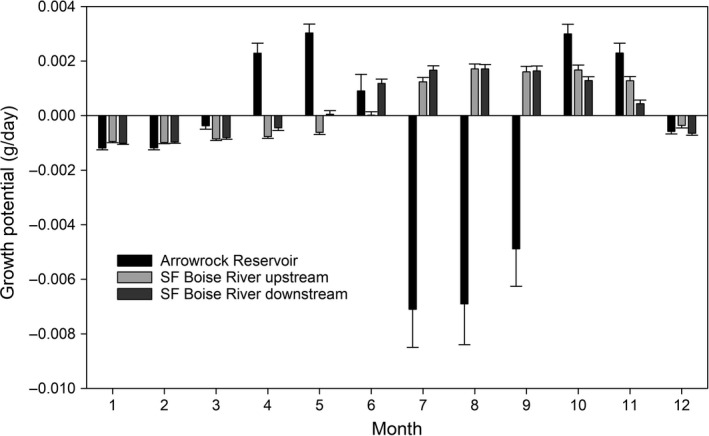
Average monthly growth potential (g/day) of female bull trout in Arrowrock Reservoir and SF Boise River using modeled average temperature (see Figure 3) and mean consumption rates (plus *SD*; see text)

The growth potential (g/day) of adult female bull trout in the study area varied by month and the areas occupied in Arrowrock Reservoir and SF Boise River (Figure 4). In Arrowrock Reservoir, potential growth was the greatest during late spring through early summer months (April–June) and in late fall (October–November), because of the influence of water temperature on metabolism. Other months suggested negative growth potential in July through September. Both the MF arm and SF arm of the reservoir were similar in monthly growth potential. In the SF Boise River, growth potential peaked in the summer (June–August) and fall (September—November) months and was negative in the remainder of the year.

**Figure 5:**
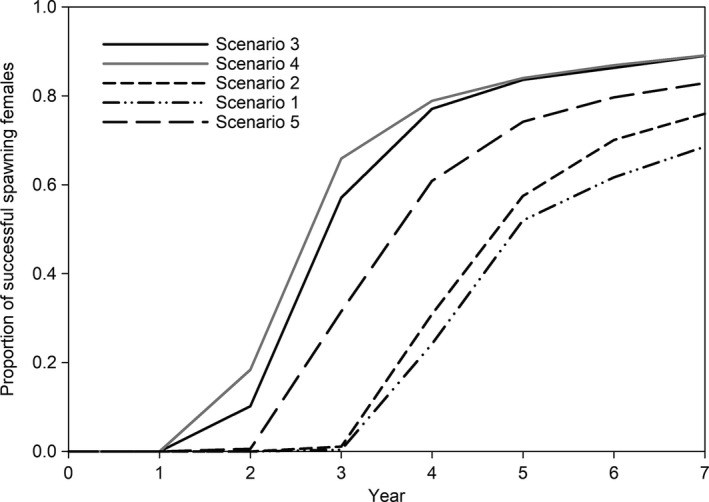
Cumulative proportion of first time of successful spawning female bull trout (*n* = 1,000) for each movement scenario based on sufficient energy content (4 kJ/g) estimated with a bioenergetics model. Year 0 is the start of model simulations with all fish 250 mm total length. See Table 1 for movement scenario descriptions

Model simulations suggest 250 mm female fish that begin migrations to Arrowrock Reservoir and the SF Boise River take two or more years to accrue enough energy to begin spawning migrations (Figure 5). Simulations also indicated that approximately 70%–90% of the female bull trout can successfully spawn within 7 years. Fish that used the reservoir during fall through early spring and the SF Boise River in the spring and summer were capable of spawning earlier and had a greater cumulative proportion of spawners compared with other movement patterns. In contrast, fish that resided solely in either of the SF Boise River locations began spawning at year four and exhibited a lower cumulative proportion of total spawning events.

Three pathways for spawning behavior were simulated: (a) migration and spawning, (b) migration and no spawning, and (c) no migration or spawning. Depending on the movement scenario, between 23% and 67% of the fish would spawn in a given year (Table 2). Most of these were fish that did not spawn in the previous year (54%–99%). In contrast, female bull trout that used Arrowrock Reservoir had up to 25% of females spawning over consecutive years. There was no consecutive repeat spawning for fish that exclusively occupied the SF Boise River.

**Table 2:** Percent of female bull trout under each movement scenario that did not migrate and spawn (skip), migrated and did not spawn (migrate), and migrated and spawned (spawn) in a spawning year relative to the decision in the previous year

Scenario	Decision	Spawning year *i*											
		4			5			6			7		
		Skip	Migrate	Spawn	Skip	Migrate	Spawn	Skip	Migrate	Spawn	Skip	Migrate	Spawn
1	Skip *i*−1	1.9	0.6	12	0.2	0.1	36	0	0	21.4	0.3	0	32.5
	Migrate *i*−1	0	20.4	11.5	0	16.1	7.8	0	13.7	4.5	0	11	5.4
	Spawn *i*−1	34.4	2.9	16.3	21.2	2	16.6	32.8	2.7	24.9	23.8	1.6	25.4
2	Skip *i*−1	0.7	0.3	14.6	0	0.1	39.7	0	0	14	0.1	0.1	38
	Migrate *i*−1	0	20.1	9	0	16	6.7	0.1	13	5.6	0	10.8	5.3
	Spawn *i*−1	39.1	2.3	13.9	14	2.6	20.9	38.1	3.1	26.1	17.9	2	25.8
3	Skip *i*−1	53.5	18.8	23.8	10.6	18	25.7	0.9	6.6	30.2	3.5	8.1	28.1
	Migrate *i*−1	0.4	3	0.1	3.2	16.4	2.2	10.9	21.2	2.3	9.2	16.2	2.4
	Spawn *i*−1	0.4	0	0	23.9	0	0	27.9	0	0	32.5	0	0
4	Skip *i*−1	34.1	17.6	29.1	7.4	2.5	26	3.3	3.1	38.6	7.3	3.1	25.3
	Migrate *i*−1	0.7	16.7	0.7	7.8	25	1.5	4.9	20.8	1.8	3.3	18.3	2.3
	Spawn *i*−1	1.1	0	0	29.8	0	0	27.5	0	0	40.4	0	0
5	Skip *i*−1	11.3	7.7	17.4	3.7	2.4	26.7	2.7	0.9	30.6	2.1	0.5	30.5
	Migrate *i*−1	0.1	19.9	12.3	0	19.6	9.1	0.5	17.2	6.2	0.2	15.7	3
	Spawn *i*−1	21.4	1.1	8.8	30.5	1.9	6.1	29.9	0.8	11.2	33.9	1.1	13

Note

See Table 1 for scenario descriptions.

**Figure 6:**
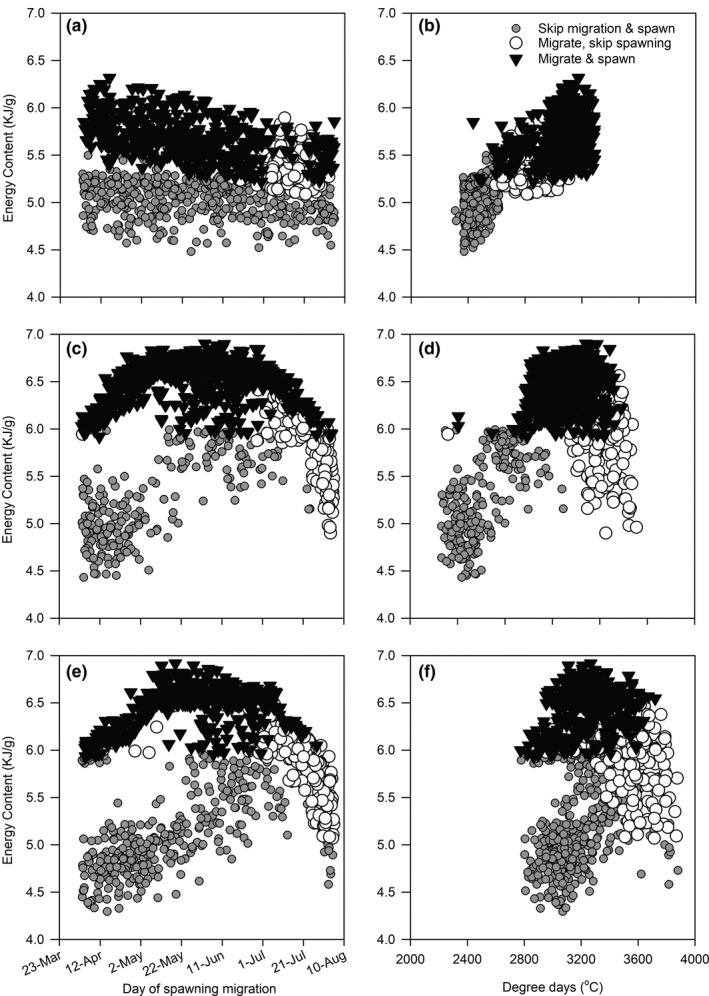
Relationship between energy content (kJ/g) prior to migration and day of spawning migration (a, c, e) or the annual degree days (°C; b, d, f) and the outcome if a fish will skip, migrate, and/or spawn for movement scenarios 1 (a, b), 3 (c, d), and 5 (e, f). See Table 1 for movement scenario descriptions

Model simulations suggest spawning female bull trout generally accrue more mass and energy before spawning compared with fish that skip spawning (Figure 6). Moreover, on average, 30% (±3% *SD*) of energy can be lost postspawning by spawning females. In contrast, fish that migrate but do not spawn lose 10% (±5%) of energy on average, whereas those fish that do not migrate or spawn gain on average 16% (±3%).

**Figure 7:**
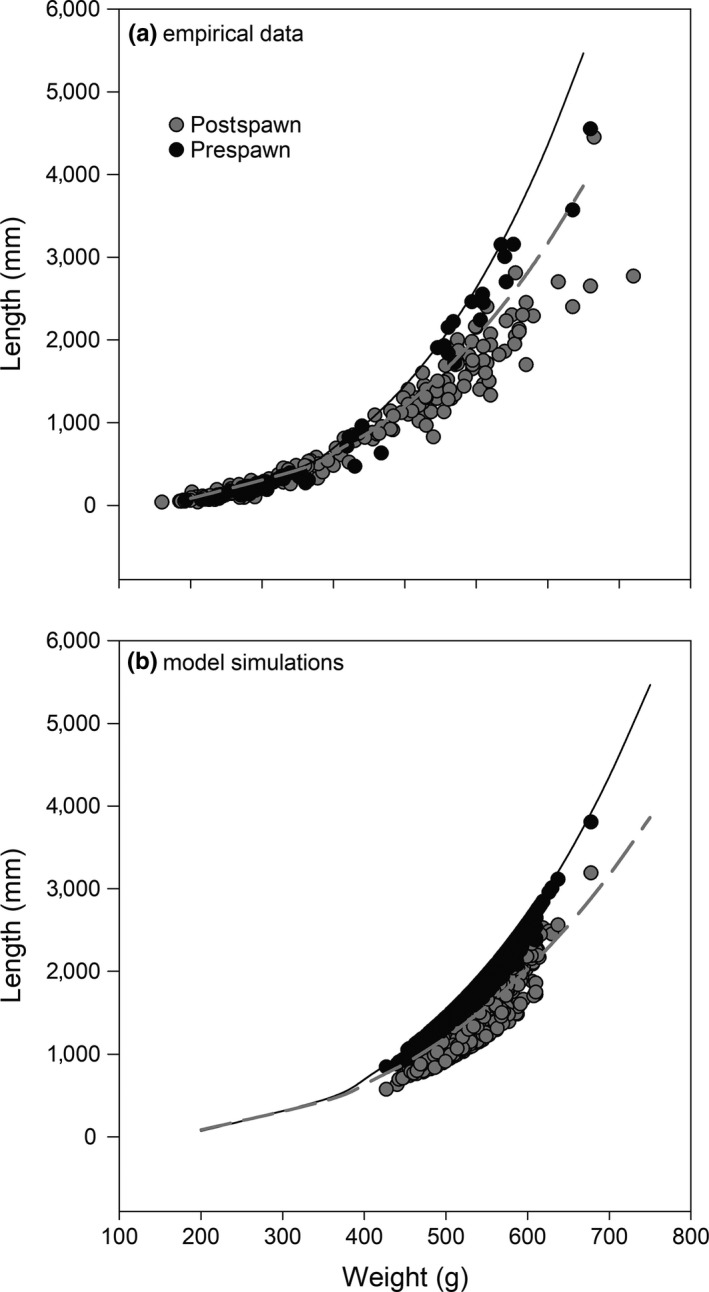
Length–weight relationship for fish captured in Arrowrock Reservoir prior to spawning (prespawn) and at a weir in the Middle Fork Boise River after spawning (postspawn; a) compared to length–weight relationship for pre‐ and postspawn fish from model simulations (b). The regression line is the same between plots a and b and was created using empirical data of captured fish. Weir location is the same as the temperature site in the Middle Fork Boise River (see Figure 2)

Although we were not able to compare model simulations with empirical data in regard to the energy density of fish pre‐ and postspawning, we could compare the length–weight relationship. Field observations suggest fish captured after spawning during September and October lose approximately 25% of their mass relative to fish captured before spawning during April and May (Figure 7a). Model simulations are consistent with the field observations (Figure 7b).

Skipped spawning was prevalent among the fish in our simulations. Regardless of the movement scenario, between 20% and 50% of the individuals did not migrate or spawn (i.e., skip; Table 2). In addition, another 20%–30% of the fish migrated but then lacked the energy reserves to spawn. Fish that skipped spawning typically spawned the following year. Most of the migrants that did not spawn began migration mid‐July or later and experienced more annual degree days compared to the trajectories displayed by other fish (Figure 5). Depending on the movement scenario, up to 18% of the migrants would never spawn during our simulations.

**Figure 8:**
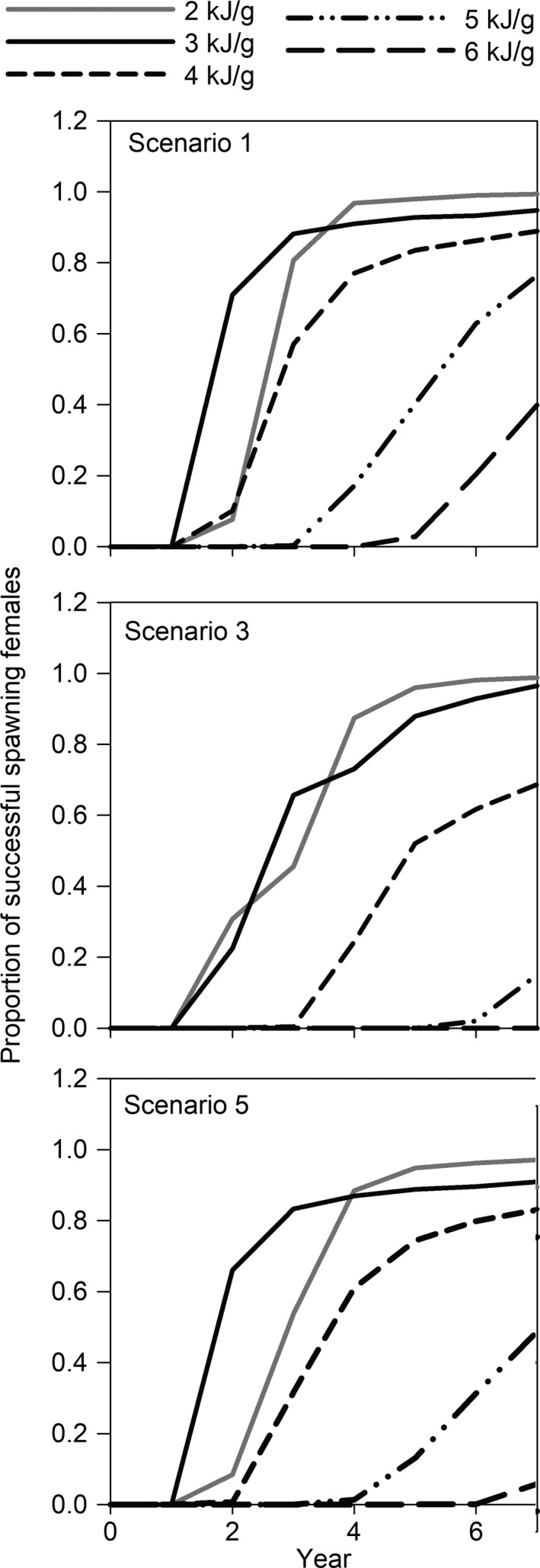
Cumulative proportion of first‐time successful spawning female bull trout (*n* = 1,000) for movement scenarios 1 (top), 3 (middle), and 5 (bottom) based on energy content threshold (2–6 kJ/g). Year 0 is the start of model simulations with all fish at 250 mm. See Table 1 for movement scenario descriptions

Changes in the energy threshold required for female bull trout to successfully migrate and spawn altered the time to maturity and proportion of individual capable of spawning (Figure 8). Compared to the energy threshold of 4 kJ/g, by lowering it to 2 or 3 kJ/g fish were capable of first spawning earlier in years and in greater numbers. Increasing the threshold to 5 or 6 kJ/g had fish taking more years to reach the energetic needs to spawn, and a smaller proportion, if at all, would spawn. Fish that only used the SF Boise River were more sensitive to changes in energy threshold values compared with fish that occupied Arrowrock Reservoir.

## 4 DISCUSSION

This study represents the first attempt to link bull trout migration behavior in a thermally heterogeneous riverscape with consequences for net energy gains and influences on age at reproduction and probability of annual reproduction. Study of each of these processes in isolation, for example, movement (e.g., Muhlfeld & Marotz, 2005; Paragamian & Walters, 2011), growth and bioenergetics (Mesa et al., 2013; Selong, McMahon, Zale, & Barrows, 2001), or thermal heterogeneity (e.g., Benjamin et al., 2016; Isaak et al., 2010), provides the foundation for our simulations to evaluate how they interact across the riverscape to influence bull trout. This approach allowed us to use thermal constraints across space and time to understand the growth, energy accumulation, and spawning potential of bull trout in the SF Boise River, which is regulated by water release from Anderson Ranch Dam, and Arrowrock Reservoir that is a focal destination for most migratory bull trout (MacCoy et al., 2017; Monnot et al., 2008). Based on energy content of female bull trout, model simulations suggest that skipped spawning may be as prevalent as annual repeat spawning for migrant fish. This is because fish often skipped if spawning occurred the previous year. However, the frequency of repeat or skipped spawning was dependent on the movement patterns and the associated thermal regimes experienced. For example, fish simulated with cooler or warmer water typically skipped spawning owing to lower condition or energy reserves.

Previous research has shown that bull trout have strong behavioral and physiological responses to their habitat, including temperature and prey availability (Eckmann et al., 2018; Gutowsky et al., 2017; Selong et al., 2001). Our study complements these by exploring how movement patterns and associated temperatures can influence growth potential across a heterogeneous river–reservoir system. Periods with the greatest growth potential for bull trout in Arrowrock Reservoir and SF Boise were consistent with timing of occupancy observed during telemetry studies. For Arrowrock Reservoir, model simulations suggest the highest growth potential was in spring and fall, which is primarily when bull trout were observed in the reservoir (Monnot et al., 2008; Maret & Schultz, 2013). In contrast, the greatest growth potential for bull trout in the SF Boise River was in summer and fall, again consistent with habitat use by fish revealed through telemetry observations (Flatter, 2000). Bull trout were rarely observed in Arrowrock Reservoir during the summer owing to increased temperatures and potentially low dissolved oxygen (Maret & Schultz, 2013), which can prevent migration through the reservoir in August and September. The pattern of moving into tributaries in the spring or early summer is consistent with other populations of bull trout (Al‐Chokhachy & Budy, 2008; Johnston & Post, 2009; Weigel et al., 2017), as well as other salmonids (Benjamin, Wetzel, Martens, Larsen, & Connolly, 2014; Young, 2011).

Similar to estimated growth potential, bull trout that moved between Arrowrock Reservoir and SF Boise River had a greater opportunity to accumulate energy, which allowed potential for more frequent spawning attempts. In contrast, individuals simulated only in the SF Boise River required more time to achieve enough energy to spawn, which negated repeat spawning over subsequent years. The patterns simulated were consistent irrespective of the energy threshold considered. Regardless of the movement patterns, our simulations suggest that migratory subadults take 2–3 years to achieve enough energy for a first spawning attempt. This pattern is consistent with other bull trout populations (Al‐Chokhachy & Budy, 2008; Fraley & Shepard, 1989) where age at first spawning migration is approximately 5 years. As a reminder, our simulated fish first migrated downstream at 250 g or about age 2. In addition, our simulations suggest the majority of female bull trout (between 70% and 90%) in the upper Boise River watershed can achieve sufficient energy to spawn at least once. However, our results do not account for potential mortality that fish may experience.

Along with the high propensity of female bull trout to spawn, our model simulations also suggest that skipped spawning can be prevalent—up to 50% of female bull trout in a given year. Previous studies observed that about 30%–50% of female bull trout may skip spawning (Fraley & Shepard, 1989; Johnston & Post, 2009), which is consistent with other iteroparous fish where up to 60% of the population may skip spawning (Rideout & Tomkiewicz, 2011; Secor, 2008). Factors influencing available energy and spawning tactics include spawning behavior the previous year, habitat conditions, and individual size (Bull & Shine, 1979; Rideout et al., 2005; Secor, 2008). However, understanding these factors in the same individual over time is logistically difficult with empirical studies. Our model estimated about 20%–40% of the female bull trout would skip spawning if they spawned the previous year. This is because bull trout in our model lost between 30% and 50% of their energy during spawning, consistent with empirical studies of other iteroparous fish (Dutil, 1986; Glebe & Leggett, 1981; Jonsson, Jonsson, & Hansen, 1991), and because of the weight loss of bull trout captured after spawning within the upper Boise River. Habitat conditions the bull trout experienced in the model influenced the duration to regain the energy lost. Typically, individuals associated with cooler temperatures had slower growth and energy gains and would skip spawning the next year. In contrast, fish that experienced warmer water may regain enough energy to migrate the following year but lack the energy reserves to spawn owing to a higher physiological demand as temperatures increase. Density dependence has also been suggested to influence skipped spawning in bull trout owing to competition for limited resources (Johnston & Post, 2009) or delaying migration timing that could restrict a spawning event (Sinnatamby et al., 2018). Further modeling efforts could link bioenergetics to population dynamics to explore how density dependence, and other factors, may influence skipped spawning.

Average temperatures used in the simulations were based on empirical data over a 1‐ to 7‐year period. Although realistic, they may not depict the actual thermal regime a bull trout may occupy (Benjamin et al., 2016). For example, the temperature we used for occupancy in the MF Boise River may not accurately depict the thermal regime experienced during spawning. However, databases at finer spatial resolution are limited to only summer temperatures (e.g., Isaak et al., 2017). Given these constraints, the daily temperature regime bull trout would experience was missed in our simulations. A bioenergetics modeling framework that worked at finer temporal scales (e.g., hourly) may provide more accurate results. However, this was beyond the scope of this study.

Bull trout may shift among thermal heterogeneous locations for various reasons such as predator avoidance, prey availability, or maximum growth (Selong et al., 2001; Gutowsky et al., 2017; but see Howell et al., 2010). Recently hypothesized is that bull trout may occupy colder temperatures to improve gamete viability (Eckmann et al., 2018) or minimize metabolic costs (Armstrong & Bond, 2013). Temperature is an important driver in reproductive development and egg viability in female fish (Jobling, Johnsen, Pettersen, & Henderson, 1995; Rideout et al., 2005). For example, occupancy in warmer waters months before spawning can delay ovulation and lower the survival of eggs (Jobling et al., 1995; Pankhurst & King, 2010), as well as reduce the investment to reproductive development (Plumb et al., 2014). Alternatively, when resources are low, bull trout may seek cooler temperatures to act as a physiological refuge. For example, Dolly Varden (*S. malma*) in Alaska can adaptively regulate assimilative capacity from pulsed resources by minimizing movement and organ (e.g., stomach and liver) function for long periods of time (Armstrong & Bond, 2013). The examples above that may influence thermal preference are likely not mutually exclusive and will require further study to fully understand.

The interaction between water temperature and regulated discharge can influence the behavior, growth, and spawning potential of fish (Plumb, 2018; Rand et al., 2006). This interaction can be more apparent in heterogeneous river–reservoir systems like the one used for our model simulations (Brekke et al., 2009; Johnson et al., 2004). Although regulated discharge was not directly incorporated within the model, it was accounted for indirectly via water temperatures used in simulations. Within the upper Boise River basin, reservoir conditions and dam operations can influence energy accumulation and migratory behaviors. For example, the volume of water in Arrowrock Reservoir begins to decrease in early July during the upstream spawning migration of bull trout, which was approximately the time when the simulated bull trout had negative growth and were more likely to skip spawning. In addition to thermal constraints, lower reservoir levels can result in reduced dissolved oxygen levels (Maret & Schultz, 2013), as well as increased predation and potential for physical barriers in the varial zone (i.e., transition between river and reservoir; Prisciandaro, 2015). In contrast, cool water release from Anderson Ranch Dam is expected to benefit thermal and physical habitat for bull trout in the SF Boise River (Benjankar et al., 2018). Our simulations suggest growth potential may benefit bull trout in the summer and fall but could result in bull trout requiring more time to regain energy lost from spawning relative to fish that move among habitats.

The modeling framework employed here is well established, but there are some assumptions worth considering in terms of how they may influence the findings reported here and in reference to implications for future research. First, the energy threshold for successful spawning to occur may vary among individuals within a population. A threshold of 4 kJ/g is commonly identified for many salmonids (Crossin et al., 2004; Jonsson et al., 1991), including congeners of bull trout (Dutil, 1986), and other iteroparous fish (Glebe & Leggett, 1981). We explored a range of potential thresholds that suggest the relative patterns remain consistent. Second, the *p*‐value we estimated from empirical growth data may be low, in part, because we did not have data on diet contents at a finer temporal scale, which is desirable (Elliot & Persson, 1978). It is more likely fish exhibit a range of consumption opportunities (Armstrong & Schindler, 2011). To account for this, we randomly allocated a daily *p*‐value (ranging from 0% to 50%), which fell within the range observed for bull trout and similar species (25%–68%; Armstrong & Schindler, 2011). Third, the amount of energy allocated to reproduction in our model (17.1%; Armstrong & Bond, 2013; Finstad et al., 2002) may not accurately reflect older fish. For example, older lake trout reduce the amount of energy investment toward somatic growth and allocate more toward reproductive investment (Plumb et al., 2014). It is uncertain whether bull trout are similar in this regard.

Overall, results of this work point to the importance of thermal heterogeneity in stream networks and how contrasting patterns of seasonal movement and migration influence the prevalence of skipped spawning in bull trout. In the river–reservoir system that we simulated, water temperatures are highly regulated (Benjankar et al., 2018; Monnot et al., 2008). In the future, temperatures throughout the Boise River basin are projected to increase (Isaak et al., 2010). Accordingly, consideration of future scenarios for stream temperature, and opportunities to manage thermal regimes in the headwaters and reservoirs may help to identify climate adaptation measures to benefit bull trout (e.g., Benjankar et al., 2018; Weigel et al., 2017). Furthermore, incorporation of all life stages and habitat use throughout the watershed via a demographic model (e.g., Jørgensen et al., 2006) may further elaborate how this species is truly influenced by changing temperatures in time and space. To date, most models of the response of bull trout to thermal heterogeneity in river networks are based on behavior (Gutowsky et al., 2017; Howell et al., 2010), growth (Eckmann et al., 2018; Selong et al., 2001), or changes in distribution associated with warming temperatures (Benjamin et al., 2016; Eby, Helmy, Holsinger, & Young, 2014; Isaak, Young, Nagel, Horan, & Groce, 2015). As results of this work highlight, consideration of skipped spawning can provide important new insights on how bull trout or other coldwater species will respond to thermal heterogeneity.

## CONFLICT OF INTEREST

None declared.

## AUTHOR CONTRIBUTION

Joseph R. Benjamin: Conceptualization (equal); data curation (equal); formal analysis (lead); funding acquisition (equal); methodology (lead); writing – original draft (lead); writing – review & editing (equal). Dmitri T. Vidergar: Conceptualization (equal); data curation (equal); formal analysis (supporting); investigation (equal); methodology (supporting); writing – original draft (supporting); writing – review & editing (equal). Jason B. Dunham: Conceptualization (equal); formal analysis (supporting); funding acquisition (equal); methodology (supporting); writing – original draft (supporting); writing – review & editing (equal).

## Data Availability

Geodatabase containing data is used in this study: ScienceBase https://doi.org/10.5066/F7MG7MQJ.
